# Minimal ATP‐Independent N_2_‐Reducing Systems Defined by L‐Cluster‐Bound Nitrogenase Assembly Platforms

**DOI:** 10.1002/anie.2968123

**Published:** 2026-04-27

**Authors:** Robert Quechol, Yimo Yang, Chi Chung Lee, Markus W. Ribbe, Yilin Hu

**Affiliations:** ^1^ Department of Molecular Biology and Biochemistry University of California Irvine California USA; ^2^ Department of Chemistry University of California Irvine California USA

**Keywords:** ATP‐independent catalysis, L‐cluster, NifB, NifEN, nitrogenase

## Abstract

The Mo‐nitrogenase, which consists of a reductase component (NifH) and a catalytic component (NifDK), catalyzes ATP‐dependent reduction of N_2_ to NH_3_ at its active‐site M‐cluster ([(*R*‐homocitrate)MoFe_7_S_9_C]). A complex metallocofactor, the M‐cluster is assembled through NifB‐mediated formation of the intermediate L‐cluster ([Fe_8_S_9_C]), followed by L‐to‐M cluster maturation on NifEN. Here, we show that the L‐cluster intrinsically endows the assembly proteins NifB and NifEN with N_2_‐reducing activity. Such a function is strictly dependent on the L‐cluster, as NifB acquires N_2_‐reducing capability only after conversion of the precursor K‐cluster (2x[Fe_4_S_4_]) to an L‐cluster. Both L‐cluster‐bound NifB (NifB^L^) and NifEN (NifEN^L^) catalyze ATP‐independent N_2_ reduction in vitro when supplied with a chemical reductant or photoexcited quantum dots. Moreover, these L‐cluster‐containing proteins support in vivo N_2_‐fixation in NifH‐deficient *E. coli* strains, where the low‐potential ferredoxin YfhL serves as an essential physiological electron donor. The intrinsic reactivity of the L‐cluster toward N_2_ supports an evolutionary model in which primordial nitrogenase was a simpler, one‐component, NifEN^L^‐like enzyme that preceded the modern, high‐efficiency two‐component system; whereas the shared L‐cluster topology found in ancient nondiazotrophic enzymes like methyl‐CoM reductase and methylthio‐alkane reductase further implies that the L‐cluster may represent an evolutionary link among nitrogen, carbon, and sulfur biogeochemical cycles.

## Graphical abstract

Association of the L‐cluster with the nitrogenase assembly proteins NifEN (NifEN^L^) or NifB (NifB^L^) intrinsically endows these proteins with N_2_‐reducing activity, enabling in vitro N_2_‐reduction by NifEN^L^ and NifB^L^ when supplied with chemical reductants or photoexcited quantum dots while supporting in vivo N_2_‐fixation in NifEN^L^‐ and NifEN^L^‐expressing *Escherichia coli* strains where the low‐potential ferredoxin YfhL serves as the electron donor.

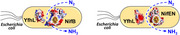

## 1 Introduction

**FIGURE 1:**
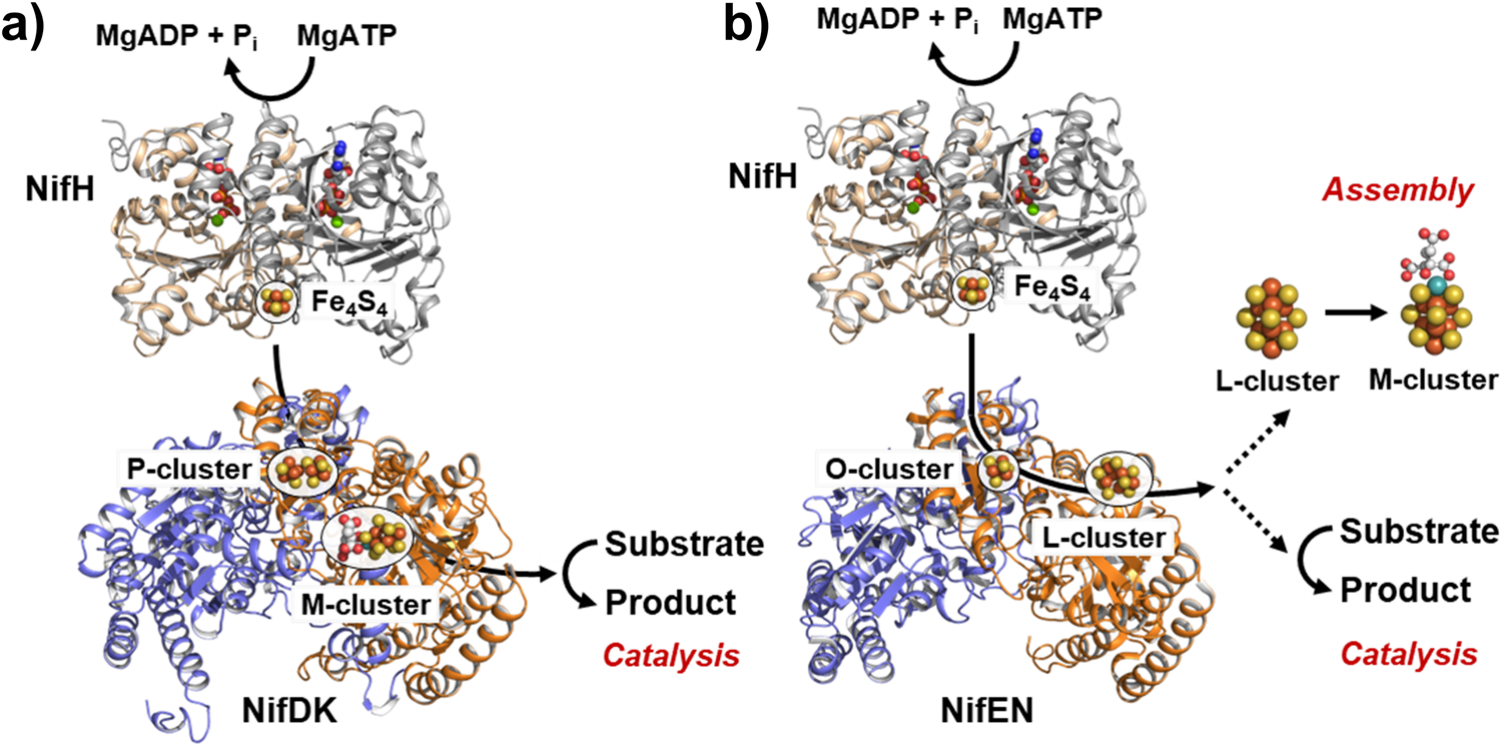
The homologous NifH/NifDK and NifH/NifEN systems. (a) The Mo‐nitrogenase is a two‐component, ATP‐dependent enzyme system composed of a reductase component (NifH) and a catalytic component (NifDK). Electrons are transferred from the [Fe_4_S_4_] cluster of NifH, through the intermediary P‐cluster ([Fe₈S₇]), to the active‐site M‐cluster [(*R*‐homocitrate)MoFe₇S₉C] of NifDK to enable substrate reduction. (b) A two‐component homolog to the Mo‐nitrogenase consists of NifH and NifEN—a structural and functional homolog to NifDK. Electrons are routed from the [Fe_4_S_4_] cluster of NifH, via the intermediary O‐cluster ([Fe_4_S_4_]), to the L‐cluster ([Fe₈S₉C]) of NifEN in an ATP‐dependent process. Such electron transfer drives either (*i*) Mo‐ and homocitrate‐dependent L‐to‐M cluster maturation (“assembly”) or (*ii*) direct substrate reduction at the L‐cluster in the absence of Mo and homocitrate (“catalysis”). For the purpose of simplicity, only one half of the tetrameric NifDK or NifEN is shown. The NifH subunits are shown in gray and light wheat; and the α‐ and β‐subunits of NifDK and NifEN are colored orange and blue, respectively. MgADP•AlF_4_^−^ (a nonhydrolyzable ATP analog) and metalloclusters are depicted as space‐filling models. The atoms are colored as follows: Fe, orange; S, yellow; Mo, cyan; O, red; C, gray; N, blue; Mg, dark green; Al, light green; F, light blue. PyMOL was used to generate this figure, using PDB entries 1N2C, [3] 3U7Q, [5] and 3PDI [8].

Nitrogenase is responsible for biological nitrogen fixation, a crucial step in the global nitrogen cycle in which the atmospheric N_2_ is converted to the bioavailable NH_3_ at ambient conditions [1, 2]. A binary enzyme system, the Mo‐dependent nitrogenase from a soil bacterium, *Azotobacter vinelandii*, comprises a reductase component and a catalytic component. The reductase component, Fe protein (NifH), is a γ_2_‐dimer with its two subunits each housing a MgATP binding site and bridged together by a [Fe_4_S_4_] cluster. The catalytic component, MoFe protein (NifDK), is an α_2_β_2_‐tetramer containing a pair of highly complex metalloclusters per αβ‐dimer: an electron‐mediating P‐cluster ([Fe_8_S_7_]), which is located at the α/β‐subunit interface; and an active‐site M‐cluster (also known as the cofactor or FeMoco; [(*R*‐homocitrate)MoFe_7_S_9_C]), which is situated within the α‐subunit (Figure 1a) [3, 4, 5, 6, 7, 8]. The two components are believed to undergo repeated association and dissociation during catalysis, which facilitates an ATP‐dependent electron transfer from the [Fe_4_S_4_] cluster of NifH, through the P‐cluster, to the M‐cluster of NifDK to enable substrate reduction (Figure 1a) [2, 3, 4, 9, 10]. Such a two‐component system is highly versatile in small‐molecule activation, capable of reducing a wide range of substrates, including N_2_, H^+^, N_3_^−^, CO, CO_2_, and C_2_H_2_ (Figure 1a) [1, 11, 12, 13].

**FIGURE 2:**
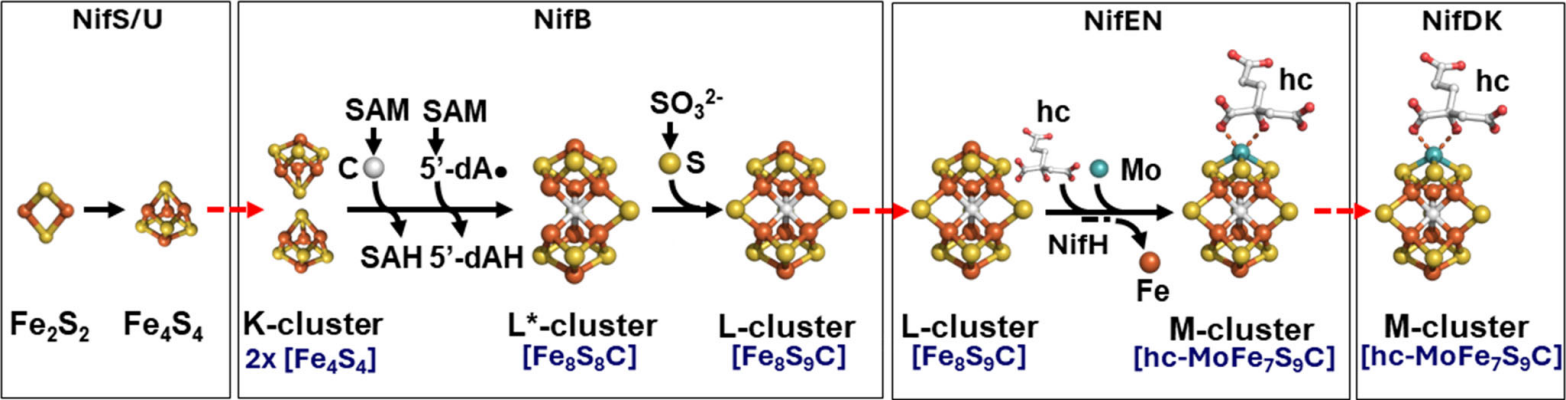
Biosynthesis of the M‐cluster. This process [12, 14, 15] is initiated with the sequential NifS/U‐mediated assembly of [Fe_2_S_2_] and [Fe_4_S_4_] clusters, followed by transfer of a [Fe_4_S_4_] cluster pair (K‐cluster) to NifB, where it is converted to a [Fe₈S₉C] cluster (L‐cluster) via radical SAM‐dependent insertion of an interstitial carbide along with the incorporation of a sulfite‐derived “9^th^” belt sulfide. The L‐cluster is then delivered to NifEN, where NifH‐dependent insertion of Mo and homocitrate (hc) enables maturation of the L‐cluster into an M‐cluster ([(*R*‐homocitrate)MoFe₇S₉C]). Finally, the fully matured M‐cluster is transferred from NifEN to its target binding site in NifDK. The metalloclusters and subunits are colored as depicted in Figure 1.

The catalytic versatility of nitrogenase is largely owing to the unique reactivity of its active‐site M‐cluster. Composed of [MoFe_3_S_3_] and [Fe_4_S_3_] partial cubanes that are connected by three bridging μ_2_ sulfido ligands (also referred to as “belt sulfides”) and one μ_6_ central carbide, the M‐cluster is one of the most complex metallocofactors found in biological systems. The assembly of the M‐cluster occurs *ex situ* through the combined actions of a series of assembly proteins (Figure 2) [12, 14, 15]. The initial stage of this process involves the synthesis of small [Fe_4_S_4_] building blocks by NifS, a cysteine desulfurase, and NifU, an FeS assembly protein [12, 14, 15]. Subsequently, a pair of [Fe_4_S_4_] clusters is delivered to NifB, a radical *S*‐adenosyl‐L‐methionine (SAM) enzyme, where the paired [Fe_4_S_4_] clusters (designated K‐cluster) are coupled and rearranged into an [Fe_8_S_9_C] cluster (designated L‐cluster) concurrently with the insertion of a SAM‐derived central carbide and a sulfite‐derived “9th” belt sulfide [8, 16, 17, 18]. The L‐cluster is then transferred to NifEN, a cofactor maturase, where it is matured into an M‐cluster upon substitution of one terminal Fe atom with Mo/homocitrate by NifH [19, 20, 21]. This event is followed by the transfer of the M‐cluster from NifEN to its final binding site within a P‐cluster replete, yet cofactor‐deficient apo‐NifDK, resulting in the formation of a P‐ and M‐cluster replete holo‐NifDK (designated NifDK^M^) [12, 22].

Structurally nearly indistinguishable from the metal‐sulfur core of the mature cofactors (i.e., the M‐, V‐, and Fe‐clusters) in the three homologous nitrogenases (i.e., the Mo, V, and Fe‐only nitrogenases) [12, 23, 24, 25], the L‐cluster can be viewed as a homocitrate‐free, Fe‐only variant of the nitrogenase cofactors [8, 26, 27, 28]. The striking resemblance to the nitrogenase cofactors and, most notably, the conservation of the subcubane‐bridging μ_2_ sulfido ligands (or belt sulfides) that are proposed to be involved in catalysis [23, 29, 30], points to the possibility of the L‐cluster mimicking the cofactors of nitrogenase in substrate reduction. Consistent with this suggestion, our recent studies revealed that the L‐cluster‐bound NifEN protein (designated NifEN^L^) was capable of in vitro [31] and in vivo [32] reduction of N_2_ to NH_3_ when paired with NifH—the ATP‐dependent reductase partner of the M‐cluster bound (designated NifDK^M^)—into a two‐component mimic (i.e., NifH/NifEN^L^) of the Mo‐nitrogenase (i.e., NifH/NifDK^M^) (Figure 1b). The ability of NifEN^L^ to substitute for NifDK^M^ in supporting substrate reduction could be ascribed to a high degree of homology in the primary sequences and tertiary structures of these two α_2_β_2_‐tetrameric proteins, as well as the presence of an analogous cluster pair at similar locations within their homologous frameworks: an O‐cluster ([Fe_4_S_4_]; NifEN^L^) or a P‐cluster ([Fe_8_S_7_]; NifDK^M^) at the α/β‐subunit interface, and an L‐cluster ([Fe_8_S_9_C]; NifEN^L^) or an M‐cluster ((*R*‐homocitrate)MoFe_7_S_9_C]; NifDK^M^) in the α‐subunit (Figure 1b) [8, 31, 32]. Such a similarity would permit an ATP‐dependent electron flow from the [Fe_4_S_4_] cluster of NifH, through the O‐cluster, to the L‐cluster in NifEN^L^ to enable substrate reduction at the active‐site L‐cluster (Figure 1b).

Interestingly, our recent studies also demonstrated the ability of NifEN^L^ to reduce N_2_ to NH_3_ in an in vitro NifH/ATP‐independent reaction driven by a strong reductant, Eu^II^‐DTPA [31, 32]. This observation is exciting, as it points to a plausible evolutionary scenario in which the ancient nitrogenase (i.e., the primitive nitrogenase enzyme that evolved the capability to catalyze the ambient reduction of dinitrogen to ammonia) was a one‐component, NifEN^L^‐like enzyme that directly accepted electrons from electron donors (e.g., ferredoxins) for substrate reduction, and that this ancient enzyme ultimately evolved into a two‐component system that used a specific ATPase, NifH, as an obligate electron donor for the catalytic NifDK component to facilitate N_2_ reduction at a much higher efficiency. Given the nonspontaneous nature of the formation of a complex, L‐cluster‐like structure, it is conceivable that the NifEN^L^‐like primitive nitrogenase was preceded by an earlier precursor enzyme—one resembling modern NifB in catalyzing the radical SAM‐dependent synthesis of an L‐cluster–like cofactor. Such a cofactor, featuring a six‐coordinate interstitial carbide to maintain structural integrity, could allow its labile belt sulfides to undergo dynamic exchange during catalysis, thereby facilitating substrate binding and product release [30, 31, 32, 33]. In this context, it is crucial to examine whether the L‐cluster endows NifB (as NifB^L^) with the ability to reduce N_2_, just as it does NifEN (as NifEN^L^), which could lend strong support to our proposed scenarios of nitrogenase evolution and functionality.

Here, we report a study that traces the N_2_‐reducing activity to the L‐cluster associated with NifB^L^ and NifEN^L^. Using a combination of biochemical, analytical, and spectroscopic analyses, we demonstrate the in vitro reduction of N_2_ to NH_3_ by NifB^L^ or NifEN^L^ in an ATP‐independent reaction driven by either a chemical reductant (Eu^II^‐DTPA) or visible light‐activated CdS@ZnS (CZS) core‐shell quantum dots (QDs). Moreover, using our recently developed heterologous expression system in *Escherichia coli*, we show in vivo ^15^N_2_ fixation by a NifH‐free, yet YfhL‐replete NifEN^L^‐ or NifB^L^‐expressing strain through nanoSIMS analysis. Our results highlight the necessity of having the core structure of L‐cluster in place to enable N_2_ reduction while illustrating a clear impact of the protein scaffold on the reactivity of the L‐cluster, thereby providing important insights into the evolution and reactivity of nitrogenase.

## 2 Results

### 2.1 In Vitro N_2_ Reduction by L‐Cluster Bound NifB and NifEN

**FIGURE 3:**
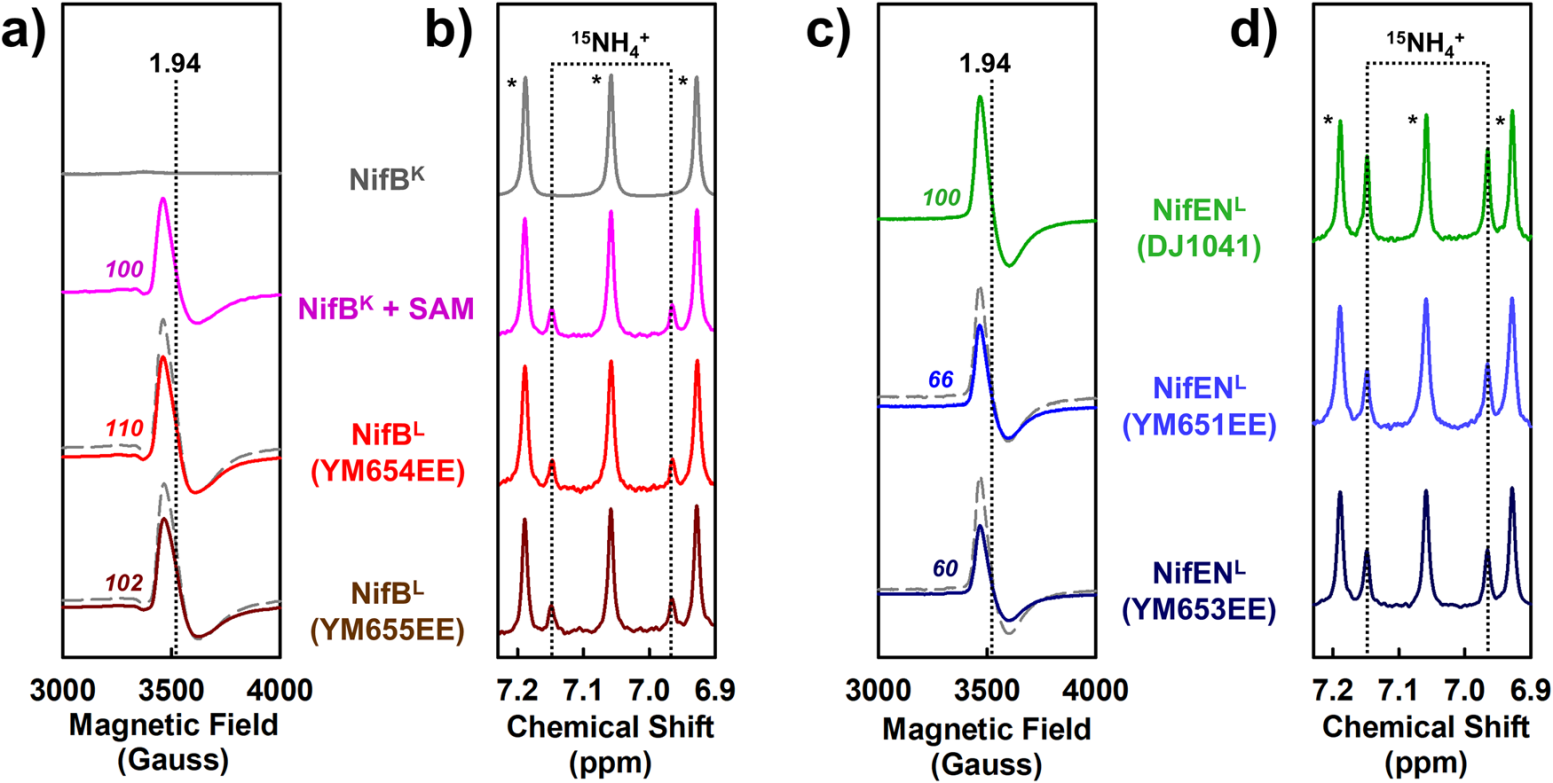
Spectroscopic and activity analyses of various NifB and NifEN species. (a) Perpendicular‐mode EPR spectra of reconstituted NifB^K^ (*gray*), reconstituted/SAM‐treated NifB^K^ (i.e., converted to NifB^L^; *pink*), and as‐isolated NifB^L^ from the *yfhL*‐replete *E. coli* strain YM654EE (*red*) and the *yfhL*‐depleted strain YM655EE (*brown*), recorded in the IDS‐oxidized state. (b) Frequency‐selective pulse ^1^H NMR spectra of Eu^II^‐DTPA‐driven ^15^N_2_‐reduction by reconstituted NifB^K^ (*gray*), reconstituted/SAM‐treated NifB^K^ (i.e., converted to NifB^L^; *pink*), and as‐isolated NifB^L^ from *yfhL*‐replete YM654EE (*red*) and *yfhL*‐depleted YM655EE (*brown*). (c) Perpendicular‐mode EPR spectra of as‐isolated NifEN^L^ from *A. vinelandii* strain DJ1041 (*green*), the *yfhL*‐replete *E. coli* strain YM651EE (*blue*), and the *yfhL*‐depleted strain YM653EE (*dark blue*) in the IDS‐oxidized state. (d) Frequency‐selective pulse ^1^H NMR spectra of Eu^II^‐DTPA‐driven ^15^N_2_‐reduction by as‐isolated NifEN^L^ from *A. vinelandii* DJ1041 (*green*), *yfhL*‐replete YM651EE (*blue*), and *yfhL*‐depleted YM653EE (*dark blue*). The *g* value of the diagnostic *g* = 1.94 feature of the oxidized L‐cluster is indicated (a, c). The protein samples are of the same concentration (a,b and c,d). The EPR traces normalized on the basis of Fe contents are shown in gray dashed lines and overlaid with the corresponding non‐normalized traces, and the relative signal intensities (expressed in %; shown as numbers above traces) were calculated by setting the signal intensity of NifB^K^+SAM or NifEN^L^ (DJ1041) as 100% (a, c). The ^15^NH_4_+‐specific doublet at ∼6.97 and ∼7.12 ppm indicates N_2_ reduction to NH_3_, whereas the triplet marked with an asterisk (*) corresponds to the natural‐abundance ^14^NH_4_+ background (b, d). The turnover numbers (TONs) are as follows: (b) NifB^K^, 0; NifB^K^+SAM, 0.4 ± 0.1; NifB^L^ (YM654EE), 0.4 ± 0.1; NifB^L^ (YM655EE), 0.4 ± 0.1; (d) NifEN^L^ (DJ1041), 3.5 ± 0.6; NifEN^L^ (YM651EE), 2.6 ± 0.4; NiEN^L^ (YM653EE), 2.5 ± 0.4.

To examine if and when NifB could acquire the ability to reduce N_2_, we prepared two forms of NifB that represented different biosynthetic stages of the Fe‐only cofactor core: (*i*) an L‐cluster bound form of NifB (NifB^L^), isolated from an *E. coli* strain (YM654EE) co‐expressing the *Methanosarcina acetivorans nifB*,*S3,U3* genes, which enabled assembly of the RS ([Fe_4_S_4_]) and K (2x[Fe_4_S_4_]) clusters on NifB and in vivo K‐to‐L cluster conversion driven by the endogenously produced SAM; and (*ii*) a K‐cluster bound form of NifB (NifB^K^), prepared by first removing clusters from NifB using the iron chelator, bathophenanthroline, and subsequently reconstituting the cluster‐deficient NifB with synthetic [Fe_4_S_4_] clusters in the absence of SAM [17]. Showing the expected subunit composition (Figure S1a), the as‐isolated NifB^L^ from YM654EE was comparable in the metal content and biosynthetic competence to the SAM‐treated NifB^K^, wherein the K‐cluster was converted to an L‐cluster (Figure S1b,c). Moreover, like the SAM‐treated NifB^K^ (Figure 3a, *pink*), the as‐isolated NifB^L^ (Figure 3a, *red*) displayed a *g* = 1.94 electron paramagnetic resonance (EPR) signal characteristic of the L‐cluster in the indigodisulfonate (IDS)‐oxidized state [18], further verifying the presence of an L‐cluster in this protein species. Commonly used as a diagnostic marker of L‐cluster formation, the near‐isotropic *g* = 1.94 signal is likely associated with an *S* = 1/2 state arising from the mixed‐valence, antiferromagnetically coupled Fe_8_ core of the L‐cluster; however, the precise electronic origin of this feature is yet to be fully elucidated.

Notably, as observed for the SAM‐treated NifB^K^ (Figure 3b, *pink*), the as‐isolated NifB^L^ (Figure 3b, *red*) was capable of reducing ^15^N_2_ to ^15^NH_3_ in an ATP‐independent, Eu^II^‐DTPA‐driven assay, as indicated by the appearance of the ^15^NH_4_^+^‐specific doublet at 6.97 and 7.15 ppm in the frequency‐selective ^1^H pulse nuclear magnetic resonance (NMR) spectrum. When supplied with visible light‐activated CdS@ZnS (CZS) core‐shell quantum dots (QDs) as alternative reducing equivalents, NifB^L^ could also support photoenzymatic reduction of ^15^N_2_ to ^15^NH_3_ as a CZS:NifB^L^ biohybrid at a higher efficiency (Figure S2a). The observation that NifB acquires the N_2_‐reducing activity only *after* the K‐to‐L cluster conversion is important, as it provides strong, albeit indirect proof for the mandatory requirement of the unique cofactor core structure and, particularly, its architecture‐stabilizing interstitial C atom and catalysis‐facile belt‐S^2−^ ions, for N_2_ reduction. Moreover, it lends solid support to the hypothesized appearance of a radical SAM‐dependent, NifB‐like L‐cluster synthase as the precursor to an L‐cluster‐bound, NifEN‐like predecessor nitrogenase.

With respect to NifEN, an L‐cluster bound form of NifEN (NifEN^L^) could be readily isolated from an *E. coli* strain (YM651EE) coexpressing the *A. vinelandii nifE,N* genes with the *M. acetivorans nifS3,U3,B* genes, in the absence of the *A. vinelandii nifH,M* genes required for the heterologous synthesis of NifH [32]. Such a “stand‐alone” NifEN^L^ (i.e., expressed without NifH) demonstrated the expected subunit composition and the comparable metal content and biosynthetic competence to the native protein from *A. vinelandii* (Figure S1d–f). Moreover, like its native counterpart (Figure 3c, *green*), NifEN^L^ displayed the L‐cluster specific *g* = 1.94 EPR signal in the IDS‐oxidized state (Figure 3c, *blue*) [32]. As observed for NifB^L^, this NifEN^L^ species not only reduced ^15^N_2_ to ^15^NH_3_ in an ATP‐independent, Eu^II^‐DTPA‐driven assay (Figure 3d, *blue*), but also supported QDs‐enabled, light‐driven reduction of ^15^N_2_ to ^15^NH_3_ at an increased efficiency as a CZS:NifEN^L^ biohybrid (Figure S2b). Collectively, the observations that the L‐cluster specifically confers the N_2_‐reducing activity upon NifEN and NifB—the only extant *nif*‐encoded proteins known to bind this cluster—point to a possible relevance of these proteins to nitrogenase evolution and functionality.

### 2.2 In Vivo N_2_ Fixation by *E. coli* Strains Expressing L‐Cluster Bound NifB and NifEN

Having established the in vitro N_2_ reduction by NifEN^L^ and NifB^L^, we then asked the question of whether these proteins could perform N_2_ fixation under in vivo conditions. To address this question, we performed nanoscale secondary ion mass spectrometry (nanoSIMS) analyses of ^15^N assimilation by the NifH‐free, NifB^L^‐ or NifEN^L^‐expressing strain (YM654EE or YM651EE) in comparison with the nitrogenase‐free control strain (MY21) under ^15^N_2_‐fixing conditions. Specifically, we cultivated the *E. coli* strains with 2 mM externally supplied NH_4_^+^, induced protein expression with 0.5 mM IPTG upon ∼50% consumption of NH_4_^+^ in the growth medium, and continued to grow cells under ^15^N_2_ for 12 h prior to harvesting, fixing, and drying the cells for nanoSIMS analyses by a CAMECA nanoSIMS 50L instrument. This previously established protocol [32, 34] would allow the use of ∼50% externally supplied NH_4_^+^ to drive pre‐induction accumulation of cell mass and biosynthetic machinery, followed by the use of the remaining ∼50% NH_4_^+^ in the growth medium to support post‐induction synthesis of nitrogenase until the cells accumulate enough nitrogenase to sustain N_2_ fixation on their own. Thus, if our one‐component, “stand‐alone” NifB^L^ or NifEN^L^ could sustain in vivo N_2_ reduction, we would observe a post‐induction elevation of ^15^N assimilation in the NifB^L^‐ or NifEN^L^‐expressing *E. coli* strain relative to that in the nitrogenase‐free control strain.

**FIGURE 4:**
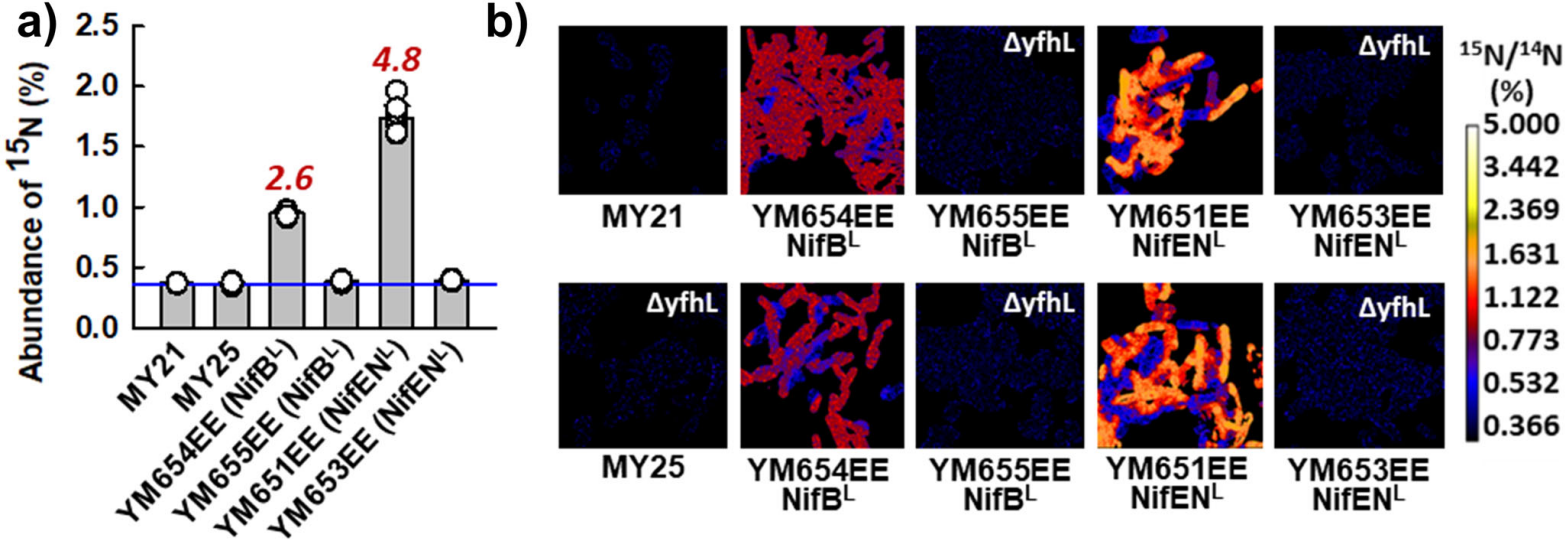
Diazotrophic nitrogen assimilation by *E. coli* expressing NifB^L^ or NifEN^L^. (a) Statistical analysis of secondary ion images derived from (b) nanoSIMS experiments of six *E. coli* strains grown under 100% ^15^N_2_: MY21, a nitrogenase‐free, *yfhL*‐replete strain; MY25, a nitrogenase‐free, *yfhL*‐depleted strain; YM654EE, a NifB^L^‐expressing, *yfhL*‐replete strain; YM655EE, a NifB^L^‐expressing, *yfhL*‐depleted strain; YM651EE, a NifEN^L^L‐expressing, *yfhL*‐replete strain; YM653EE, a NifEN^L^‐expressing, *yfhL*‐depleted strain. The ^15^N abundance of each strain was calculated based on data collected in 6 different regions of interest (ROI) of the nanoSIMS images of the samples shown in the figure. The average values of ^15^N abundance (^15^N/^14^N) are: MY21 (n = 1), 0.37% ± 0.01%; MY25 (n = 1), 0.36% ± 0.01%; YM654EE (n = 2), 0.95% ± 0.02%; YM655EE (n = 2), 0.38% ± 0.01%; YM651EE (n = 2), 1.74% ± 0.1%; YM653EE (n = 2), 0.39% ± 0.01%. Data are expressed as mean ± s.d., and individual data points are indicated as open circles in a. Given the natural ^15^N abundance (^15^N/^14^N) of 0.37% (indicated by a blue line in panel a), the ^15^N enrichment levels are 2.6‐fold and 4.8‐fold, respectively, for YM654EE and YM651EE.

Indeed, statistical analyses performed on six different regions of interest (ROI) within the secondary ion image of each sample revealed the average level of ^15^N enrichment, or ^15^N/^14^N ratio, of 0.37% ± 0.01% for the nitrogenase‐free MY21 control, which was identical to the natural abundance ^15^N/^14^N ratio of 0.37%; in contrast, they illustrated average ^15^N/^14^N ratios of 0.95% ± 0.02% and 1.74% ± 0.1%, respectively, for YM654EE (expressing NifB^L^) and YM651EE (expressing NifEN^L^), reflecting ^15^N enrichment levels of 2.6‐ and 4.8‐fold, respectively, relative to the natural‐abundance background (Figure 4a,b). The higher ^15^N incorporation level of the NifEN^L^‐expressing strain (YM651EE) than that of the NifB^L^‐expressing strain (YM654EE) could very well reflect a closer resemblance of NifEN to the catalytic NifDK component of the extant nitrogenase; moreover, it points to a distinct modulating effect of protein scaffold on the N_2_‐reducing activity of the active‐site L‐cluster.

### 2.3 Electron Donor for N_2_ Reduction by L‐Cluster Bound NifB and NifEN

The observation that NifEN^L^ and NifB^L^ could enable in vivo N_2_ fixation on their own was exciting, yet it begged the question of what acted as the physiological electron donor for these L‐cluster‐bound proteins in the cell, given that no exogenous ferredoxin or flavodoxin was introduced into the *E. coli* strains that heterologously expressed NifB^L^ (YM654EE) and NifEN^L^ (YM651EE). Previously, we demonstrated that deletion of YfhL, an endogenous two‐cluster ferredoxin of *E. coli* with a high degree of sequence homology (47%) to the *A. vinelandii* two‐cluster ferredoxin FdxN, abolished the in vivo N_2_‐reducing activity of the two‐component nitrogenase analog (NifH/NifEN) [32]. Additionally, we provided theoretical and experimental evidence for the interaction and electron transfer between YfhL and NifH, as well as an increased in vitro N_2_‐reducing activity of the NifH/NifEN analog upon incubation with increasing amounts of pre‐reduced YfhL [32]. These results are consistent with the low reduction potential of YfhL ([Fe_4_S_4_] cluster 1: *E* ^0^’ = −675 mV; [Fe_4_S_4_] cluster 2: *E* ^0^’ = −418 mV) [35], which makes this ferredoxin well suited to function in place of FdxN in delivering electrons to NifH (*E* ^0^’ = −405 mV) [36]. Given the requirement of low‐potential electrons to drive the challenging N_2_ reduction at the L‐cluster, it is likely that the in vivo N_2_ fixation mediated by the NifH‐independent, one‐component system (i.e., NifB^L^ or NifEN^L^) is likewise supported by a low‐potential ferredoxin, such as YfhL. Consistent with this notion, YM655EE (expressing NifB^L^) and YM653EE (expressing NifEN^L^)—two strains identical to YM654EE and YM651EE except for carrying the *yfhL*‐deletion—did not accumulate ^1^ ^5^N above the natural‐abundance ^1^ ^5^N/^14^N background of the nitrogenase‐free, *yfhL*‐deletion control strain, MY25 (Figure 4a,b). This finding indicates no measurable incorporation of ^1^ ^5^N by YM655EE and YM653EE from labeled N_2_ under these conditions; moreover, it strongly implicates YfhL as an essential physiological donor for L‐cluster‐based N_2_ fixation under in vivo conditions.

**FIGURE 5:**
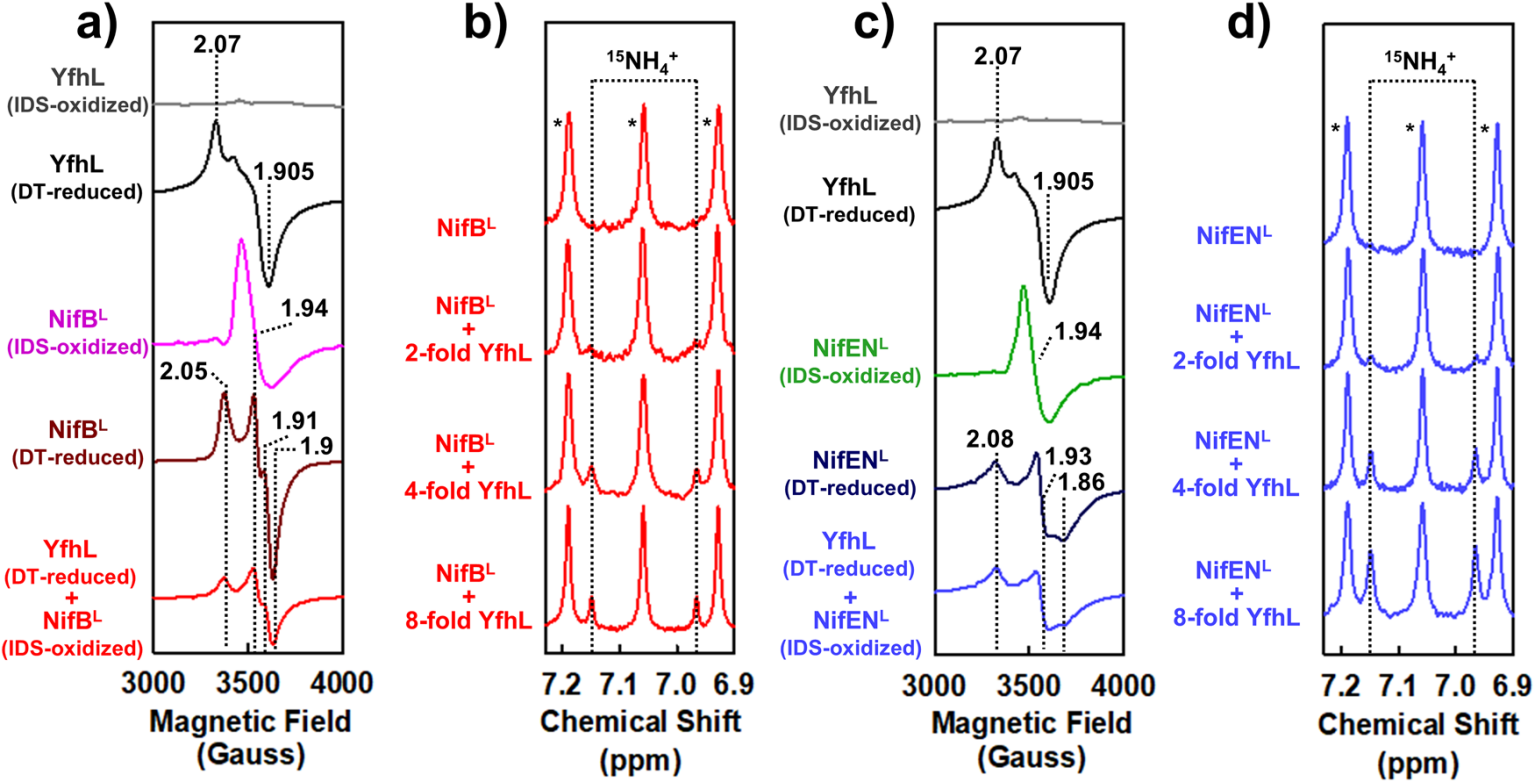
Interaction between YfhL and NifB^L^ or NifEN^L^. (a) EPR analysis of electron transfer between YfhL and NifB^L^. Shown are the perpendicular‐mode EPR spectra of YfhL (*gray*, *black*) or NifB^L^ (*pink*, *brown*) in IDS‐oxidized and DT‐reduced states, and a mixture of reduced YfhL and oxidized NifB^L^ after 1 h incubation (*red*). The transfer of electrons from reduced YfhL to oxidized NifB^L^ is indicated by the disappearance of features characteristic of reduced YfhL, concomitant with the appearance of features characteristic of reduced NifB^L^. (b) Frequency‐selective pulse ^1^H NMR spectra of Eu^II^‐DTPA‐driven ^15^N_2_‐reduction by NifB^L^ alone and in the presence of twofold, fourfold, and eightfold molar excess of reduced YfhL. (c) EPR analysis of electron transfer between YfhL and NifEN^L^. Shown are the perpendicular‐mode EPR spectra of YfhL (*gray*, *black*) or NifENL (*green*, *dark blue*) in IDS‐oxidized and dithionite‐reduced states, and a mixture of reduced YfhL and oxidized NifEN^L^ after 1 h incubation (*blue*). The transfer of electrons from reduced YfhL to oxidized NifEN^L^ is indicated by the disappearance of features characteristic of reduced YfhL, concomitant with the appearance of features characteristic of the reduced NifEN^L^. (d) Frequency‐selective pulse ^1^H NMR spectra of Eu^II^‐DTPA‐driven ^15^N_2_‐reduction by NifEN^L^ alone and in the presence of twofold, fourfold, and eightfold molar excess of reduced YfhL. The *g* values are indicated in the EPR spectra (a, c). The ^15^NH_4_+‐specific doublet at ∼6.97 and ∼7.12 ppm indicates N_2_ reduction to NH_3_, whereas the triplet marked with an asterisk (*) corresponds to the natural‐abundance ^14^NH_4_+ background (b, d). The TONs are as follows: (b): NifB^L^ alone, 0; NifB^L^+twofold YfhL, 0.1 ± 0.02; NifB^L^+fourfold YfhL, 0.3 ± 0.07; NifB^L^+eightfold YfhL, 0.4 ± 0.1; (d) NifEN^L^ alone, 0; NifEN^L^L+twofold YfhL, 0.2 ± 0.04; NifEN^L^+fourfold YfhL, 1.1 ± 0.2; NifEN^L^+eightfold YfhL, 2.2 ± 0.4. IDS, indigodisulfonate; DT, dithionite.

**FIGURE 6:**
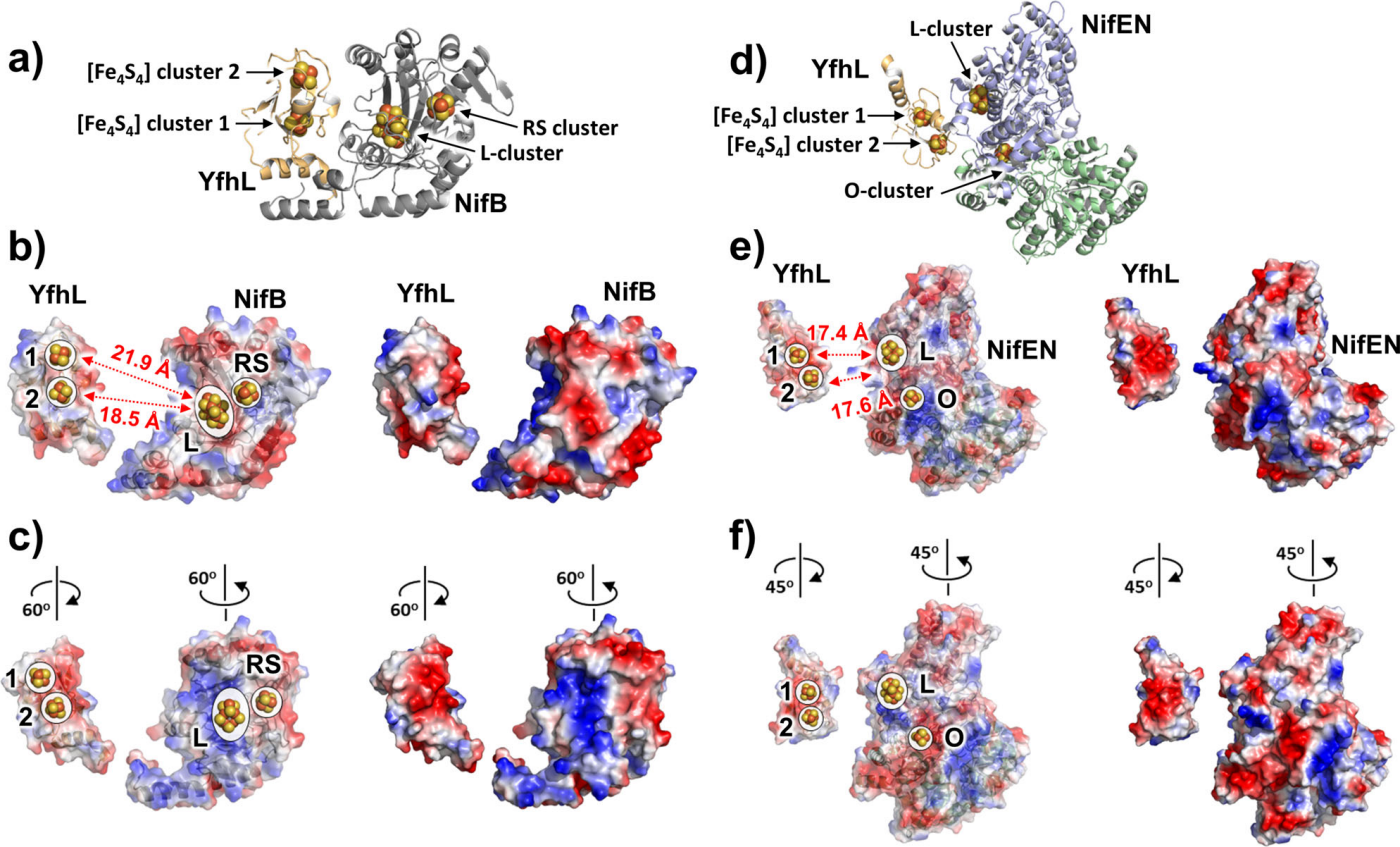
Interaction of YfhL with NifB and NifEN. The Boltz‐2 [38, 39] predicted models of (a–c) YfhL/NifB and (d–f) YfhL/NifEN complexes are shown as (a,d) ribbon and (b,c,e,f) surface presentations. Predictions were guided using PDB entries 2ZVS (YfhL), [35] 7JMB (NifB), [40] and 3PDI (NifEN) [8] as structural templates. In panels a and d, YfhL, NifB, and the α‐ and β‐subunits of NifEN are depicted in orange, grey, blue, and green, respectively. In panels b, c, e, and f, the negative and positive charges are indicated in red and blue, respectively. The images on the left use a transparent surface to mark the relative position of metal‐sulfur clusters, while the images on the right use a solid rendering to highlight surface charge distribution. Clusters are displayed in ball‐and‐stick presentations, with Fe and S atoms colored orange and yellow, respectively. The models show high confidence, scoring 0.82 (panels b–c) and 0.84 (panels d–f) overall. Confidence in the predictions is further supported by low RMSD values—0.551 Å (YfhL, panels a–c), 0.541 Å (YfhL, panels d–f), 0.669 Å (NifB, panels a–c), and 0.578 Å (NifEN, panels d–f)—when compared with the available x‐ray crystallographic structures.

It is worth noting that NifB^L^ and NifEN^L^ purified from the *yfhL*‐deletion strains YM655EE and YM653EE remained L‐cluster‐replete (Figure 3a, *brown*; Figure 3c, *dark blue*) and retained the ability to reduce N_2_ in vitro in ATP‐independent, Eu^II^‐DTPA‐driven assays (Figure 3b, *brown*; Figure 3d, *dark blue*). This observation aligns well with the previously reported, disparate redox requirements for nitrogenase assembly and catalysis [37], with assembly enabled by a weaker reductant at a more positive reduction potential (e.g., flavodoxins) and catalysis facilitated by a stronger reductant at a more negative reduction potential (e.g., YfhL). More importantly, this finding indicates that the loss of in vivo N_2_‐fixing ability in YM655EE or YM653EE stems from the absence of YfhL as a low‐potential electron donor, rather than from any defect in the structural integrity or L‐cluster content of NifB^L^ and NifEN^L^. Additional support for this argument came from studies of the interactions between purified YfhL and NifB^L^ or NifEN^L^. EPR analysis indicated an electron transfer from the reduced YfhL to the oxidized NifB^L^ or NifEN^L^, as evidenced by the disappearance of the oxidized L‐cluster‐specific signal in NifB^L^ (Figure 5a, *red vs. pink*) or NifEN^L^ (Figure 5c, *blue vs. green*) and the concurrent appearance of features characteristic of reduced NifB^L^ (Figure 5a, *red vs. brown*) or NifEN^L^ (Figure 5c, *blue vs. dark blue*), accompanied by the disappearance of the reduced [Fe_4_S_4_]^+^ cluster‐specific signal in YfhL upon mixing with NifB^L^ (Figure 5a, *red vs. black*) or NifEN^L^ (Figure 5c, *blue vs. black*). Frequency‐selective pulse ^1^H NMR analysis revealed a clear titration effect of pre‐reduced YfhL on NH_4_^+^ formation, showing progressive increases in NH_4_^+^ production when Eu^II^‐DTPA was replaced with increasing amounts of pre‐reduced YfhL (Figure 5b,d) such that the YfhL‐driven activity ultimately reached a level comparable to that observed with Eu^II^‐DTPA (see Figure 3b,d). Together, these results establish YfhL as a physiological low‐potential electron donor capable of driving NifH/ATP‐independent N_2_ reduction by NifEN^L^ and NifB^L^ heterologously expressed in *E. coli*. Supporting this conclusion, Boltz‑2 [38, 39] based predictions indicated highly feasible interactions between YfhL and NifB (Figure 6a–c) or NifEN (Figure 6d–f). In these models, the YfhL‐bound [Fe_4_S_4_] clusters are positioned within medium‐to‐long‐range electron‐tunneling distances of the active‐site L‐clusters in both NifB and NifEN, providing structurally plausible electron transfer pathways consistent with the observed catalytic activities.

It should be emphasized that computational docking of small ferredoxins can be sensitive to orientation and may yield multiple degenerate solutions; accordingly, the Boltz‐2 model should be interpreted with caution as one of several plausible configurations. Future comparisons with alternative approaches, together with structural analyses, could further refine and validate these interaction models.

## 3 Discussion

The necessity of having the unique cofactor core structure in place to enable N_2_ reduction, as illustrated by the ability of NifB to reduce N_2_ to NH_3_ only *after* the K‐to‐L cluster transformation has taken place, again points to the cofactor belt‐sulfides as a structural/functional feature central to the nitrogenase mechanism. Linking two [M_4_S_3_] subcubanes (M = Fe, Mo, V) along with a core‐stabilizing interstitial carbide, the three labile belt‐sulfides (S3A, S2B, S5A) undergo dynamic mobilization during catalysis, prompting our proposal that N_2_ initially binds via displacement of S3A, followed by stepwise N_2_ reduction at S2B and S5A as the cluster rotates, culminating in NH_3_ release upon replacement of S5A [23, 29, 30]. Such a sequential reduction of N_2_ is presumably facilitated by the well‐evolved protein environment in the catalytic component (NifDK) of the modern‐day nitrogenase that offers disparate proton‐donating abilities to the three belt‐sulfur locations—a feature absent from the previously proposed, ancient prototype nitrogenase (a NifEN^L^‐like enzyme) and its L‐cluster synthesizing precursor (a NifB^L^‐like enzyme), wherein the three belt‐sulfurs are indiscriminate in the binding and reduction of N_2_ due to the lack of a well‐defined protein environment [31, 41]. The acquisition and increase of the N_2_‐reducing activity in the sequence of NifB^K^ (no activity) < NifB^L^ < NifEN^L^ provides strong support for this proposal, whereas a further increase of this activity in the order of NifEN^L^ < NifEN^L^/NifH < NifDK^M^/NifH implies a possible evolution from an inefficient one‐component enzyme to an efficient two‐component system comprising a specific electron donor (NifH) and an optimized active site (in NifDK) [31, 41].

Interestingly, phylogenetic and structural evidence suggest that the L‐cluster topology was also exploited by non‐nitrogen‐fixing enzymes during evolution. Specifically, the cluster was found in the methyl‐coenzyme M reductase (MCR) activation complex (FeSI, FeSII, FeSIII) in methanogenic archaea [42]. The presence of these clusters in the MCR activation machinery, which is believed to have originated in the last common ancestor of TACK, Asgard, and Euryarchaeota [42], implies that the L‐cluster could be used for MCR activation to direct low‐potential electrons to reduce the Ni(II) ion in the F430 cofactor. The recent discovery of an L‐cluster in the methylthio‐alkane reductase (MAR), a nitrogenase‐like enzyme that specializes in C‐S bond cleavage [43, 44], further supports the hypothesis that the last common ancestor of Nif and MAR contained this large active‐site cluster. While the question of whether the MCR system (or its like) predates the nitrogenase enzyme, or *vice versa*, is not fully settled, both are undisputedly ancient systems that gave rise to, or intertwined with, key metabolic milestones like methanogenesis, nitrogen reduction, sulfate reduction, and oxygenic photosynthesis during evolution.

Importantly, all L‐cluster‐dependent systems identified to date contain NifB or a homologous radical SAM enzyme, underscoring that the L‐cluster emerged alongside the rise of radical chemistry required to generate its unique topology. The exciting discoveries of the L‐cluster—first in nitrogenase (an enzyme involved in N metabolism) [45] and later in MCR (an enzyme involved in C metabolism) and MAR (an enzyme involved in C and S metabolism)—point to the L‐cluster as an evolutionary link between the major biogeochemical cycles of N, C, and S on Earth. At the same time, these observations provide strong, albeit indirect support for our proposed mechanism of N_2_ reduction that is intimately associated with the mobilization of belt‐sulfurs in a sulfite‐reductase‐like reaction [30]. Future exploration of L‐cluster–containing systems will not only clarify their evolutionary chronology but also uncover new chemistries that extend our understanding of how early life harnessed and diversified low‐potential electron transfer, thereby informing efforts to engineer novel redox catalysts and redesign L‐cluster‐like cofactors—where even the modest ATP‐independent N_2_‐reducing capacity underscores the appeal of these clusters as energy‐efficient catalytic platforms.

## Conflicts of Interest

The authors declare no conflicts of interest.

## Supporting information




**Supporting File 1**: anie72447‐sup‐0001‐SuppMat.pdf.

## Data Availability

The data that support the findings of this study are available from the corresponding author upon reasonable request.
