# Supplementary material for: Minimal ATP‐Independent N2‐Reducing Systems Defined by L‐Cluster‐Bound Nitrogenase Assembly Platforms
Source: Angew Chem Int Ed Engl. 2026 Apr 27;65(24):e2968123. doi: 10.1002/anie.2968123 (PMC13245594; doi:10.1002/anie.2968123)
Supplement: Supplementary file 1 — Supporting File 1: anie72447‐sup‐0001‐SuppMat.pdf. [file ANIE-65-e2968123-s001.pdf]

Supporting Information

Minimal ATP-independent N_2-reducing systems defined by L-cluster-bound nitrogenase assembly platforms

Robert Quechol,+[a] Yimo Yang,+[a] Chi Chung Lee,+[a] Markus W. Ribbe,*[a][b] Yilin Hu*[a]

[a]Department of Molecular Biology and Biochemistry, University of California, Irvine, CA 92697-3900
[b]Department of Chemistry, University of California, Irvine, CA 92697-2025

*Correspondence should be addressed to mribbe@uci.edu and yilinh@uci.edu
+These authors contributed equally to this work.

Experimental Section

All chemicals were purchased from Sigma-Aldrich (St. Louis, MO) and Thermo Fisher Scientific (Waltham, MA) unless specified otherwise. All experiments were conducted in a glove box or on a Schlenk line under an Ar atmosphere, with an O_2 concentration of <3 ppm.

Strain Construction

To express His-tagged $NifB^L$, genes encoding *M. acetivorans* NifS3, NifU3, and His-tagged NifB were codon-optimized for *Escherichia coli* expression, synthesized, and cloned into pRSFDuet-1 (harboring *Ma nifS3,U3,B* genes). The resulting plasmid was transformed into (*i*) *E. coli* strain MY21, which was derived from BL21(DE3) but lacked *iscR*, the gene encoding the regulator of FeS cluster biosynthesis; and (*ii*) *E. coli* strain MY25, which was identical to MY21 but lacked *yfhL*, the gene encoding the low-potential [Fe_4S_4] ferredoxin YfhL,[1] yielding strains YM654EE (from *i*) and YM655EE (from *ii*). To express His-tagged $NifEN^L$, genes encoding His-tagged *Azotobacter vinelandii* NifE and NifN, along with those encoding *M. acetivorans* NifS3, NifU3, and NifB, were codon-optimized for *E. coli* expression, synthesized, and cloned into pCDFDuet-1 (harboring *Av nifE,N* genes) and pRSFDuet-1 (harboring *Ma nifS3,U3,B* genes), respectively. The resulting plasmids were co-transformed into *E. coli* strains (*i*) MY21 and (*ii*) MY25, yielding strains YM651EE (from *i*) and YM653EE (from *ii*). Gene synthesis and cloning were carried out by GenScript (Piscataway, NJ). Other than the strains described above, *E. coli* strain YM114EE, which expressed His-tagged NifB from *Methanosarcina acetivorans*,[2] was used for generating $NifB^K$ through *in vitro* cluster reconstitution.

Cell Growth, Protein Purification and Cluster Reconstitution

E. coli strains YM114EE, YM651EE, YM653EE, YM654EE, and YM655EE were cultivated in 10-L batches of LB medium (Difco) supplemented with 50 mM MOPS/NaOH (pH 7.4), 25 mM glucose, and 2 mM ferric ammonium citrate. Cultures were grown in a BIOFLO 415 fermenter (New Brunswick Scientific) at 37°C with agitation at 200 rpm and an airflow rate of 10 L/min. Antibiotics were added as follows: 19 mg/L kanamycin for strains YM654EE and YM655EE; 19 mg/L kanamycin plus 26 mg/L streptomycin for strains YM651EE and YM653EE; and 25 mg/L chloramphenicol plus 50 mg/L ampicillin for strain YM114EE. Cell growth was monitored at OD_{600} using a Spectronic 20 Genesys spectrometer (Spectronic Instruments). When OD_{600} reached 0.5, the airflow was terminated and the fermenter was purged with N_2 (ultra-high purity) at a rate of 1.5 L/min; additionally, the temperature was lowered to 24°C. Once the temperature reached 24°C, 2 mM cysteine were added, followed by induction of protein expression with 250 μM IPTG. After 16 h of protein expression, cells were harvested by centrifugation with a Thermo Fisher Scientific Legend XTR centrifuge. The heterologously expressed His-tagged proteins were then purified by immobilized metal affinity chromatography (IMAC) as described previously.[1,2] Additionally, the NifB protein isolated from *E. coli* strain YM114EE was subjected to chelation and reconstitution procedures as described previously to yield $NifB^K$.[3]

Azotobacter vinelandii strains DJ1162, DJ1143, DJ1041 and YM9A,[4-6] which expressed His-tagged NifH, $NifB^{apo}$, $NifEN^L$ and $NifEN^{apo}$, respectively, were cultivated in 180-L batches of Burke's minimal medium supplemented with 2 mM ammonium acetate. Cultures were grown in a 200-L fermenter (New Brunswick Scientific) at 30°C with agitation at 100 rpm and an airflow rate of 30 L/min. Cell growth was monitored at OD_{436} using a Spectronic 20 Genesys spectrometer

(Spectronic Instruments). Upon depletion of ammonia, cultures were de-repressed for 3 h prior to harvesting with a flow-through centrifugal harvester (Cepa). His-tagged NifH and $NifEN^L$ were purified according to previously published procedures.[5,6]

Metal Analysis

The Fe contents of the proteins examined in this study were determined by inductively coupled plasma optical emission spectroscopy (ICP-OES) using an iCAP7000 ICP-OES instrument (Thermo Scientific). Calibration of the equipment was performed by using standard solutions made via dilution of a stock solution of elemental Fe (1 mg/mL). The protein sample was first mixed with 100 µL of concentrated sulfuric acid (H_2SO_4) and 100 µL of concentrated nitric acid (HNO_3), and subsequently heated for 30 min at 250°C. Such a procedure was repeated until the solution became colorless, followed by cooling of the solution to room temperature and dilution of the solution to a total volume of 7.5 mL with 2% HNO_3 prior to metal analysis.

M-cluster Maturation Assays

For NifEN-based maturation assays, each reaction was assembled in a total volume of 0.9 mL containing 25 mM Tris–HCl (pH 8.0), 0.4 mg of the P-cluster-containing but M-cluster-deficient $NifDK^{apo}$ (isolated from *A. vinelandii* strain DJ1143),[4,6] 1.2 mg of NifH, and 1.0 mg of $NifEN^L$ isolated from *E. coli* strain YM651EE or YM653EE, or from *A. vinelandii* strain DJ1041.[5] In addition, each reaction contained 0.4 mM homocitrate, 0.4 mM molybdate (Na_2MoO_4), 2.4 mM ATP, 4.8 mM $MgCl_2$, 30 mM creatine phosphate, 24 units of creatine phosphokinase, and 20 mM dithionite (DT; $Na_2S_2O_4$), along with 1.0 atm Ar in the headspace. For NifB-based maturation assays, each reaction was assembled in the same total volume (0.9 mL) containing 25 mM Tris–HCl (pH 8.0), 0.4 mg of the P-cluster-containing but M-cluster-deficient $NifDK^{apo}$, 1.2 mg of NifH, 1.0 mg of O-cluster-containing but L-cluster-deficient $NifEN^L$ (isolated from *A. vinelandii* strain YM9A),[5] and 2.3 mg of $NifB^L$ isolated from *E. coli* strain YM654EE or YM655EE, or 2.3 mg of $NifB^K$ treated with 10 mM SAM (*i.e.*, converted to $NifB^L$). In addition, each reaction contained 0.4 mM homocitrate, 0.4 mM Na_2MoO_4, 2.4 mM ATP, 4.8 mM $MgCl_2$, 30 mM creatine phosphate, 24 units of creatine phosphokinase, and 20 mM $Na_2S_2O_4$, along with 1.0 atm Ar in the headspace. The reaction was incubated at 30°C for 60 min and subsequently split into triplicates in three 9.5-mL vials, each containing 1.05 mg of *Av*NifH, 25 mM Tris–HCl (pH 8.0), 2.5 mM ATP, 5.0 mM $MgCl_2$, 30 mM creatine phosphate, 0.125 mg of creatine phosphokinase, and 20 mM $Na_2S_2O_4$ in a total volume of 0.7 mL, along with 0.1 atm C_2H_2 and 0.9 atm Ar in the headspace. The reaction mixture was then incubated at 30°C for 10 min. To detect C_2H_4 as a product of C_2H_2-reduction, 250 µL of the headspace was injected into a GC-FID (SRI Instruments, Torrance CA) equipped with a packed Poropak N column (Restek, Bellefonte, PA). Calibration was achieved by injecting 15 ppm C_2H_4 gas standard under the same conditions.

Light-driven assays

The synthesis of CdS@ZnS (CZS) quantum dots (QDs) and the preparation of CdS nanorods (NRs) were performed as described earlier.[7] The photocatalytic reactions of substrate reduction were assayed in 10 mL glass GC vials with a micro-stirbar. Ligand-exchanged QDs/NRs were diluted in 500 mM HEPES buffer (pH 7.2) under 1.0 atm $^{15}N_2$, followed by injection of 0.1 M sodium dithionite (anaerobically prepared in the same 500 mM HEPES buffer) and 15 µM $NifB^L$ (from YM654EE) or $NifEN^L$ (from YM651EE). The final reaction mixture contained 2 mM

$Na_2S_2O_4$ with the desired concentrations of QDs/NRs (0.5 μM) and nitrogenase protein (1.25 μM) in a total volume of 1.5 μL. In-situ formation of biohybrids occurred within 1 min, as indicated by an increased turbidity of the mixture. After incubation for 10 min in a 30 °C incubator, the mixture was irradiated at 30°C for 60 min with the purple light at 400 nm (~4.4 mW/cm^2 at sample location) using a 12 V 48-SMD LED array. Subsequently, the reaction was quenched with 0.1M H_2SO_4 and further processed for NMR analysis of $^{15}NH_4^+$ formation as described below.

Electron Paramagnetic Resonance (EPR) Experiments

Individual samples of NifB^L, NifEN^L or YfhL were prepared in a Vacuum Atmospheres glove box filled with Ar and operated at <3 ppm O_2, and flash frozen in liquid nitrogen prior to analysis. The reduced samples contained 10% (vol/vol) glycerol, 250 mM imidazole, 2 mM $Na_2S_2O_4$ and 25 mM Tris–HCl (pH 8.0) and the oxidized samples were prepared by incubating the reduced samples with excess indigodisulfonate (IDS) for 5 min. The concentration of the reduced or oxidized NifB^L, NifEN^L or YfhL samples were 15 mg/mL. To examine electron transfer from YfhL to NifB^L or NifEN^L, reduced YfhL was mixed with oxidized NifB^L (from YM654EE) or NifEN^L (from YM651EE) at a molar ratio of 1:2, yielding a total protein concentration of 25 mg/mL. The mixtures were incubated with stirring for 1 h at room temperature under an Ar atmosphere. An aliquot of 300 μL was then transferred to an EPR tube and flash-frozen prior to analysis. EPR data were acquired using an ESP 300E spectrophotometer (Bruker) interfaced with an ESR-9002 liquid-helium continuous-flow cryostat (Oxford Instruments), with a microwave power of 5 mW, a gain of 5×10^4, a modulation frequency of 100 kHz, and a modulation amplitude of 5 G. Eight scans of perpendicular-mode EPR spectra were recorded for each sample at 10 K (for the reduced samples) and 15 K (for the oxidized sample), respectively, using a microwave frequency of 9.62 GHz.

Nanoscale Secondary Ion Mass Spectrometry (NanoSIMS) Analysis

E. coli strains YM651EE and YM654EE (expressing NifEN^L and NifB^L, respectively, in a *yfhL*-replete background), YM653EE and YM655EE (expressing NifEN^L and NifB^L, respectively, in a *yfhL*-depleted background), and MY21 and MY25 (nitrogenase-free strains in a *yfhL*-replete and a *yfhL*-depleted background, respectively) were cultivated in 100 mL of supplemented M9 medium as described previously.[1] Cultures were grown for 10 h under air (until the mid-log phase) in 250-mL screw-capped Erlenmeyer flasks equipped with septum-capped side arms, after which the gas phases were exchanged with 1.0 atm $^{15}N_2$. IPTG was then added to a final concentration of 0.5 mM, and cells were grown for an additional 12 h (until the late log/early stationary phase) prior to analysis. For nanoSIMS analysis, 250-μL aliquots of the cultures (diluted to the same OD) were pipetted onto a 7 mm x 7 mm silicon square dice cut from wafers (UniversityWafer, Boston, MA) with a diameter of 2.5 cm. The samples were subsequently fixed with a PBS solution containing 4% formaldehyde for 1 h at room temperature, washed sequentially with PBS, 1:1 PBS/ethanol and ethanol, and dried on the wafer.[8] The secondary ion ($^{14}N^{12}C^-$ and $^{15}N^{12}C^-$) and secondary electron images were acquired with the CAMECA NanoSIMS 50L ion microprobe at Caltech (Pasadena, CA). A +8 keV primary Cs^+ beam of ∼1 pA was used to raster the samples in 20 × 20 μm areas. Secondary ion ($^{14}N^{12}C^-$ and $^{15}N^{12}C^-$) images of −8 keV were collected simultaneously with electron multiplier detectors. The interferences (*e.g.*, $^{13}C^{13}C^-$ to $^{14}N^{12}C^-$; $^{14}N^{13}C^-$ to $^{15}N^{12}C^-$) were fully removed from the masses of interest under the high mass resolution conditions of the

mass spectrometer. Ion images of 512×512 pixels were processed with the L'image software (http://limagesoftware.net/).

Frequency-selective Pulse Nuclear Magnetic Resonance (NMR) Analysis

The ATP-independent, Eu^{II}-DTPA-driven turnover reactions were assembled in a total volume of 1.5 mL and contained 25 mM Tris-HCl (pH 8.0), 5 mg of $NifB^{K}$ (with or without SAM), $NifB^{L}$ (from *E. coli* strain YM654EE or YM655EE) or $NifEN^{L}$ (from *A. vinelandii* strain DJ1041 or *E. coli* strain YM651EE or YM653EE), and 20 mM Eu^{II}-DTPA. Titration assays with YfhL were assembled in a total volume of 1.0 mL and contained 25 mM Tris-HCl (pH 8.0), 5 mg of $NifB^{L}$ (from *E. coli* strain YM654EE) or $NifEN^{L}$ (from *E. coli* strain YM651EE), and pre-reduced, yet reductant-free, YfhL at a molar ratio of 1:2, 1:4 or 1:8. The reaction was incubated at 30°C for 30 min under 1.0 atm $^{15}N_2$, followed by transfer of the mixture to a 1.5-mL tube containing a Microcon Centrifugal Filter (MWCO: 10 kDa; Millipore). Subsequently, the proteins were removed from the mixture by centrifugation at 10,000 g for 20 min, and 0.5 mL of the flow-through (*i.e.*, the protein-removed mixture) was combined with 0.05 mL of 1M H_2SO_4 and 0.025 mL of CD_3CN as a lock agent. The ^{1}H NMR spectra were recorded using a Bruker AvanceCore 400mHz spectrometer equipped with a CPBBO cryoprobe. Water suppression was employed, and spectra were referenced by setting the residual CH_3CN signal to 2.06 ppm.[9] A total of 512 scans were recorded per sample, with an acquisition time of 1.5 s and a relaxation delay of 5 s.

Boltz-2 Modeling

The feasibility of whether the two $[Fe_4S_4]$ cluster-containing ferredoxin YfhL could form viable complexes with NifB or NifEN was evaluated through Boltz-2 modeling,[10,11] conducted on the Tamarind Bio server (https://www.tamarind.bio/). Amino acid sequences of NifE and NifN of *A. vinelandii*, NifB of *M. acetivorans* and YfhL of *E.coli* were obtained from the JGI webpage (https://img.jgi.doe.gov/) and provided in FASTA format. The L-cluster structure was supplied as ligand SMILES ([C+2]12345[Fe]67S[Fe]18[S-]9[Fe]21S[Fe]32[S-]6[Fe]3[S-]7[Fe]4([S-]23)S[Fe]52[S-]8[Fe]9[S-]12) with defined metal coordination geometry. Template-guided structural prediction was performed by using the mmCIF files of PDB entries 2ZVS (YfhL),[12] 7JMB (NifB)[13] and 3PDI (NifEN)[14] and applying a template distance threshold of 3 Å. Boltz-2 was run in protein-ligand mode with default diffusion parameters (200 steps, step scale=1.638) and three recycling iterations. Five models were generated per run. Predicted models were analyzed for the overall confidence scores. The quality of the structure was assessed by visual inspection and through evaluation of cluster coordination geometry. The models exhibit high confidence values, with overall scores ranging from 0.82 to 0.84. Confidence in these predictions is further supported by low RMSD values—approximately 0.55 Å for YfhL, 0.67 Å for NifB, and 0.58 Å for NifEN—when compared with the available x-ray crystallographic structures. Moreover, all cluster ligands are positioned within reasonable binding distances, reinforcing the high reliability of the predicted models.

References

[1] Y. A. Liu, C. C. Lee, K. Górecki, M. T. Stiebritz, C. Duffin, J. B. Solomon, M. W. Ribbe, Y. Hu, "Heterologous synthesis of a simplified nitrogenase analog in *Escherichia coli.*" *Sci Adv.* **2025**, 11, eadw6785.

[2] A. W. Fay. J. A. Wiig, C. C. Lee, Y. Hu, "Identification and characterization of functional homologs of nitrogenase cofactor biosynthesis protein NifB from methanogens." *Proc Natl Acad Sci U. S. A.* **2015**, 112,14829.
[3] K. Tanifuji, C. C. Lee, N. S. Sickerman, K. Tatsumi, Y. Ohki, Y. Hu, M. W. Ribbe, "Tracing the 'ninth sulfur' of the nitrogenase cofactor via a semi-synthetic approach." *Nat. Chem.* **2018**, 10, 568.
[4] B. Schmid, M. W. Ribbe, O. Einsle, M. Yoshida, L. M. Thomas, D. R. Dean, D. C. Rees, B. K. Burgess, "Structure of a cofactor-deficient nitrogenase MoFe protein." *Science* **2002**, 296, 352.
[5] Y. Hu, A. W. Fay, M. W. Ribbe, "Identification of a nitrogenase FeMo cofactor precursor on NifEN complex." *Proc. Natl. Acad. Sci. U. S A.* **2005**, 102, 3236.
[6] Y. Hu, A. W. Fay, P. C. Dos Santos, F. Naderi, M. W. Ribbe, "Characterization of *Azotobacter vinelandii nifZ* deletion strains. Indication of stepwise MoFe protein assembly." *J. Biol. Chem.* **2004**, 279, 54963.
[7] Y. Ding, C. C. Lee, Y. Hu, M. R. Ribbe, P. Nagpal, A. Chatterjee, "Light-driven transformation of carbon monoxide into hydrocarbons using CdS@ZnS : VFe protein biohybrids." *ChemSusChem* **2023**, 16, e202300981.
[8] A. Pernthaler, A. E. Dekas, C. T. Brown, S. K. Goffredi, T. Embaye, V. J. Orphan, "Diverse syntrophic partnerships from deep-sea methane vents revealed by direct cell capture and metagenomics." *Proc. Natl. Acad. Sci. U. S. A.* **2008**, 105, 7052.
[9] G. R. Fulmer, A. J. M. Miller, N. H. Sherden, H. E. Gottlieb, A. Nudelman, B. M. Stoltz, J. E. Bercaw, K. I. Goldberg, "NMR chemical shifts of trace impurities: common laboratory solvents, organics, and gases in deuterated solvents relevant to the organometallic chemist." Organometallics 2010, 29, 2176.
[10] S. Passaro, G. Corso, J. Wohlwend, M. Reveiz, S. Thaler, V. R. Somnath, N. Getz, T. Portnoi, J. Roy, H. Stark, D. Kwabi-Addo, D. Beaini, T. Jaakkola, R. Barzilay, *bioRxiv* preprint **2025**, DOI: 10.1101/2025.06.14.659707.
[11] J. Wohlwend, G. Corso, S. Passaro, N. Getz, M. Reveiz, K. Leidal, W. Swiderski, L. Atkinson, T. Portnoi, I. Chinn, J. Silterra, T. Jaakkola, R. Barzilay, *bioRxiv* preprint **2025**, DOI: 10.1101/2024.11.19.624167.
[12] E. Saridakis, P. Giastas, G. Efthymiou G, Thoma, J. M. Moulis, P. Kyritsis, I. M. Mavridis, "Insight into the protein and solvent contributions to the reduction potentials of $[4Fe\text{-}4S]^{2+/+}$ clusters: crystal structures of the *Allochromatium vinosum* ferredoxin variants C57A and V13G and the homologous *Escherichia coli* ferredoxin." *J. Biol. Inorg. Chem.* **2009**, 14, 783.
[13] W. Kang, L. A. Rettberg, M. T. Stiebritz, A. J. Jasniewski, K. Tanifuji, C. C. Lee, M. W. Ribbe, Y. Hu, "X-ray crystallographic analysis of NifB with a full complement of clusters: structural insights into radical SAM-dependent carbide insertion during nitrogenase cofactor assembly." *Angew. Chem. Int. Ed. Engl.* **2021**, 60, 2364.
[14] J. T. Kaiser, Y. Hu, J. A. Wiig, D. C. Rees, M. W. Ribbe, "Structure of precursor-bound NifEN: a nitrogenase FeMo cofactor maturase/insertase." *Science* **2011**, 331, 91.

Supporting Figures

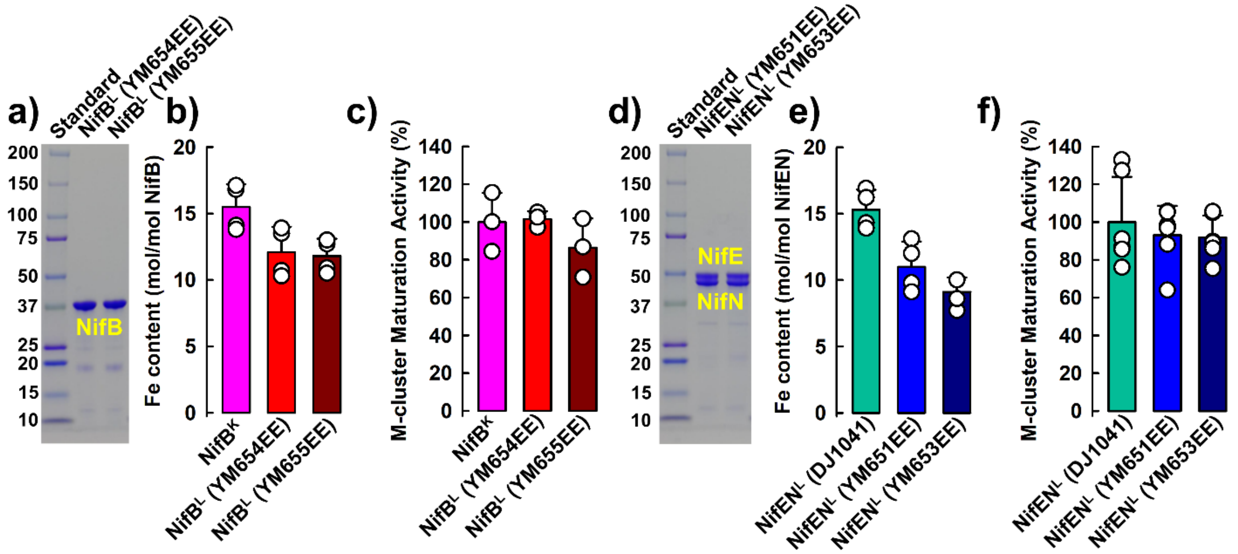

Figure S1. Biochemical characterization of various NifB and NifEN species. (**a**) SDS-PAGE analysis of as-isolated NifB^L from *yfhL*-replete *E. coli* strain YM654EE and *yfhL*-depleted *E. coli* strain YM655EE. (**b**,**c**) Maturation activities (C_2H_2-reduction to C_2H_4; b) and Fe contents (c) of reconstituted/SAM-treated NifB^K (*i.e.*, converted to NifB^L; *pink*), as-isolated NifB^L from *yfhL*-replete *E. coli* strain YM654EE (*red*) and *yfhL*-depleted *E. coli* strain YM655EE (*brown*). (**d**) SDS-PAGE analysis of as-isolated NifEN^L from *A. vinelandii* strain DJ1041, *yfhL*-replete *E. coli* strain YM651EE and *yfhL*-depleted *E. coli* strain YM653EE. (**e**,**f**) Maturation activities (C_2H_2-reduction to C_2H_4; e) and Fe contents (f) of as-isolated NifEN^L from *A. vinelandii* strain DJ1041 (*green*), *yfhL*-replete *E. coli* strain YM651EE (*blue*) and *yfhL*-depleted *E. coli* strain YM653EE (*dark blue*). Protein standard, Precision Plus Protein Kaleidoscope prestained protein standard (Bio-Rad). Data (n=3-6) are expressed as mean±s.d., and individual data points are indicated as open circles in b, c, e, and f.

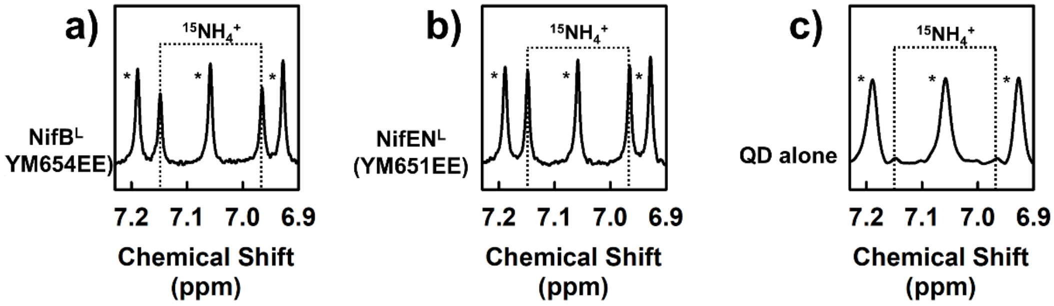

Figure S2. Quantum dots (QDs) driven $^{15}N_2$-reduction by NifB and NifEN. Frequency-selective pulse ^{1}H NMR spectra of (**a**,**b**) QDs-driven $^{15}N_2$-reduction by (a) as-isolated NifB^L from *E. coli* strain YM654EE and (b) as-isolated NifEN^L from *E. coli* strain YM651EE, and (**c**) protein-free control containing only QDs. The $^{15}NH_4^+$-specific doublet at ~6.97 and ~7.12 ppm indicates N_2 reduction to NH_3, whereas the triplet marked with an asterisk (*) corresponds to the natural-abundance $^{14}NH_4^+$ background. The turnover numbers (TONs) are as follows: (a) 1.3±0.2; (b) 4.6±0.8.
